# Markers of cardiac injury in patients with liver cirrhosis

**DOI:** 10.3325/cmj.2023.64.362

**Published:** 2023-10

**Authors:** Stjepan Šimić, Tomo Svaguša, Ivica Grgurević, Sanda Mustapić, Marko Žarak, Ingrid Prkačin

**Affiliations:** 1 Vuk Vrhovac University Clinic for Diabetes, Endocrinology and Metabolic Diseases, Merkur University Hospital, Zagreb, Croatia; 2 Department of Cardiovascular Disease, Dubrava University Hospital, Zagreb, Croatia; 3 Department of Gastroenterology, Dubrava University Hospital, Zagreb, Croatia; 4 Department of Gastroenterology, Dubrava University Hospital, Zagreb, Croatia; 5 Clinical Department of Laboratory Diagnostics, Dubrava University Hospital, Zagreb, Croatia; 6 Faculty of Pharmacy and Biochemistry, University of Zagreb, Zagreb, Croatia; 7 Department of Internal Medicine, Merkur University Hospital, Zagreb, Croatia; 8 University of Zagreb, School of Medicine, Zagreb, Croatia

## Abstract

Liver cirrhosis is an increasing public health problem and a major cause of morbidity and mortality. Accordingly, cirrhotic cardiomyopathy, a frequently underdiagnosed condition, is becoming a growing health problem. In the last 20 years, cardioselective biomarkers have been investigated for their diagnostic and prognostic properties for numerous conditions. The aim of this article is to review the literature on the relationship between the most commonly used cardioselective biomarkers (cardiac troponins I and T, N-terminal pro-B-type natriuretic peptide, brain natriuretic peptide, and heart-type fatty-acid binding protein) and the presence, functional stage, and clinical outcomes of liver cirrhosis. Elevated plasma levels of these biomarkers have been reported in patients with liver cirrhosis, and there is mounting evidence on their predictive value for clinical outcomes in this disease. In addition, elevated plasma levels of these biomarkers have been reported in patients before, during, and after liver transplantation, but in fewer studies. Due to their predictive value for clinical outcomes, we advocate the use of these markers in patients with liver cirrhosis and cirrhotic cardiomyopathy, as well as in candidates for liver transplant.

Liver cirrhosis is an increasing public health problem and a major cause of morbidity and mortality (1). The most common causes of liver cirrhosis are chronic hepatitis B and C, alcoholic liver disease, and nonalcoholic steatohepatitis (2). Liver cirrhosis is characterized by liver function deterioration and portal hypertension (PH). In the first stage of liver cirrhosis, PH occurs because of increased resistance to the flow of portal blood through the liver due to fibrosis and dysfunction of the sinusoidal endothelium, and distortion of the vascular network (3). At this stage, the disease is mostly asymptomatic, so the prevalence of compensated liver cirrhosis in the general population is underestimated (4,5). Further damage to the liver tissue reduces the liver’s synthetic, excretory, and metabolic function and increases PH. This process leads to complications such as esophageal varices (EV), splenomegaly, and hypersplenism, accumulation of ascites, spontaneous bacterial peritonitis, portal encephalopathy, and hepatorenal and hepatopulmonary syndrome (6). The severity and prognosis of liver cirrhosis are assessed by the Child-Pugh score and model for end-stage liver disease (MELD) score (7). Since serum sodium concentration is a predictor of mortality in patients with liver cirrhosis, models were developed that, in addition to standard MELD parameters, also take into account serum sodium concentration, such as MELD-Na and MELD to serum/sodium ratio (8). Central hypovolemia in patients with liver cirrhosis causes sympathetic activation, which leads to hyperdynamic circulation, and an increase in heart rate and stroke volume (9).

## Cirrhotic cardiomyopathy

Cirrhotic cardiomyopathy is a clinical entity defined in 2005 to separate the impact of liver cirrhosis on the heart from that of toxic alcoholic cardiomyopathy. Cirrhotic cardiomyopathy is a worsening of the heart function in patients with liver cirrhosis in the form of deteriorated diastolic relaxation and contractile response to stress and changes in the ECG record (prolongation of the QT interval), accompanied by hypertrophy of the left ventricle. For the diagnosis of cirrhotic cardiomyopathy, it is essential to exclude other cardiovascular diseases as the cause of cardiac dysfunction or left ventricular hypertrophy (10,11). In 2020, the Cirrhotic Cardiomyopathy Consortium proposed updated criteria for cirrhotic cardiomyopathy based on newer concepts and knowledge of heart failure. Clinical and ultrasound criteria must be met to establish a diagnosis of cirrhotic cardiomyopathy (12). Although it was previously assumed that the severity of cirrhotic cardiomyopathy correlates with the severity of the clinical presentation of liver cirrhosis (13), the results of the dobutamine stress test did not confirm this correlation (14,15).

## Markers of cardiac injury

Cardiac troponin T (cTnT) and cardiac troponin I (cTnI), together with troponin C (TnC), are proteins that bind to calcium and enable cardiac contraction (16). Due to the presence of large protein complexes of cTn in the blood, the assumed main routes of cTn clearance in myocardial infarction are endocytosis and degradation in the reticuloendothelial system. In contrast, the main route of cTn clearance in conditions that lead to its slight increase in the blood is glomerular filtration. In this case, cTn in the blood is mainly found in the form of degradation products, ie, the molecules of lower molecular weight (17,18). In addition to myocardial infarction, troponin concentrations can be elevated in many other conditions, and in some they also have prognostic significance (19). Smaller amounts of cTnI and cTnT are found as free forms in the cytosol and are responsible for their early elevation in myocardial infarction (20-22).

The newest (fifth) generation of highly sensitive cTn assays is able to determine cTn concentration in almost the entire population. hsTnI assays for TnI are produced by numerous manufacturers, while only one manufacturer produces hsTnT assays for cTnT (23). cTn are a good predictive marker for cardiovascular events (24). Their increased concentration in the blood indicated an increased ten-year cardiovascular risk (25). Elevated concentrations were observed in the healthy population, dialysis patients, and pregnant women (26). Both cTn (TnT and TnI) were positively correlated with survival in dialysis patients (27), while in the healthy population they were used to identify patients with increased cardiovascular risk (28,29).

Markers of heart failure, such as brain natriuretic peptide (BNP) and N-terminal proBNP (NT-proBNP), are secreted from the myocardium mainly as a result of myocardial stretch, although their secretion may result from the endocrine action of endothelin, angiotensin II, and adrenaline. Unlike BNP, whose clearance is mediated by the receptor for the clearance of natriuretic peptides (NPR-C) in the target organs (atria, kidneys, lungs, aorta, vein endothelium, etc), NT-proBNP is predominantly excreted by the kidney and has a much longer half-life (30-32). Plasma concentrations of BNP and NT-proBNP are clinically important in the diagnosis of heart failure (HF), in assessing the severity of the disease, in prognosis, and in evaluating treatment efficacy (31,33).

Heart-type fatty acid binding protein (H-FABP) is an intracellular protein of cardiomyocytes. It is initially detectable in plasma about 30 minutes after myocardial injury, peaks after 6-8 hours, and is excreted by glomerular filtration (34,35). In conditions of myocardial ischemia (and not necrosis), H-FABP increases, while cTn concentrations remain stable (36). H-FABP proved to be a good independent prognostic factor. It is an independent predictor of cardiovascular (CV) outcome in patients with stable coronary disease and an extremely good prognostic factor of long-term mortality in patients who have recovered from AMI (36,37)

Cardiac dysfunction is one of the main causes of mortality in patients with liver cirrhosis, both during and after liver transplantation, as well as after the placement of a transjugular porto-systemic stent shunt (TIPS) (38). Given that the liver metabolizes and excretes a large number of biological compounds, including some originating from the myocardium, their values are expected to increase in the serum of patients with liver cirrhosis. The relationship between the liver and markers of cardioselective biomarkers has not yet been fully clarified. Therefore, the primary aim of this review is to present current knowledge about the dynamics of cardiac injury markers in patients with liver cirrhosis and to define their potential clinical applications in the selection of high-risk patients. The secondary goal is to define the diagnostic value and other potential applications of individual myocardial injury markers, taking into account the specificities of patients with liver cirrhosis.

## Troponin concentrations in the blood of patients with liver cirrhosis

Elevated cTnI was found in patients with liver cirrhosis without CV disease more frequently than in healthy controls, but cTnI concentrations did not correlate with the severity of cirrhosis and its complications (39). The authors (39) assumed that elevated cTnI concentrations were associated with asymptomatic alcoholic heart damage since most of the patients suffered from alcoholic liver cirrhosis, which supports the finding of reduced LVEF (39). However, in a prospective study by Mihailovici et al, cTnI concentrations correlated with the clinical stage of liver cirrhosis according to the Child-Pugh classification and MELD score (40).

cTnT was found to be higher in patients with liver cirrhosis and was a significant predictor of the severity of liver cirrhosis as assessed by the Child-Pugh and MELD score (40-43). Also, its concentration correlated with overall mortality in patients with liver cirrhosis regardless of the presence of cardiac disease (41-46). cTnT concentration also correlated with portal vein diameter, the length of the corrected QT interval in the ECG, left ventricular mass, interventricular septal diameter, peak velocity of atrial filling, and carotid intima-media thickness (42). cTnT concentrations were significantly higher in hospitalized patients with decompensated liver cirrhosis compared with those with compensated liver cirrhosis (45). However, cTnt significantly correlated with gastrointestinal bleeding but not with other forms of cirrhosis decompensation during hospital stay (45). It has to be noted that most participants of the mentioned studies had alcoholic or post-HCV liver cirrhosis, while patients with other causes of cirrhosis were less represented (45). The relationships between cardiac troponins and mortality, Child-Pugh and MELD scores, and left ventricular function are shown in Table 1.

**Table 1:** The correlations of cardiac troponin T and I concentrations with mortality, Child-Pugh score, model for end-stage liver disease (MELD) score, and left ventricular function

	Mortality	Child-Pugh score	MELD score	Left ventricular function
**cTnI concentrations**	No corrleation	Positive correlation (40)	Positive correlation (40)	Negative correlation (39,41)
**cTnT concentrations**	Positive correlation (41,43,46)	Positive correlation (41-43)	Positive correlation (41-43)	Negative correlation (42,44,46)

*Abbreviations: cTnI – cardiac troponin I; cTnT – cardiac troponin T.

## Troponin in liver transplantation

Preoperative cTnI concentrations were found to correlate with 30-day and one-year mortality in patients after liver transplant (47). No significant correlation was established with seven-day mortality, but this finding could be interpreted with caution due to the small sample size of the study (48). Also, preoperative cTnI concentrations and the presence of cardiovascular disease before transplantation were found to correlate with graft rejection and overall mortality within one year after liver transplantation. A combination of these two factors was a better predictor than either factor alone (49). Furthermore, a combination of preoperative cTnI concentrations and BNP concentrations was a good predictor of 90-day mortality and graft loss in patients after liver transplantation (50). Intraoperatively elevated cTnT values during liver transplantation were a good predictor of postoperative 30-day mortality (51). Postoperative elevation in cTnI concentrations in patients who previously had not had elevated cTnI concentration correlated with total mortality and graft dysfunction during hospital stay (52). A postoperative increase in cTnI concentrations was a good predictor of mortality 24 hours after liver transplantation, but the main factor associated with an increase in cTnI concentrations was the operation length (53). Also, postoperative increase in cTnT concentrations correlated with acute kidney injury in patients who underwent liver transplant and did not have any underlying cardiovascular disease (54). The relationships between perioperative cardiac troponin concentrations in patients who underwent liver transplantation and mortality, graft dysfunction and rejection, and acute kidney injury are summarized in Table 2.

**Table 2:** The correlations of cardiac troponin T and I concentrations with the outcomes of liver transplantation: mortality, graft dysfunction or rejection, and acute kidney injury

	Mortality	Graft dysfunction/rejection	Acute kidney injury
**cTnI concentrtions**	Positive correlations with preoperative and postoperative concentrations (47,49,52,53)	Positive correlations with preoperative and postoperative concentrations (49,52)	No correlation
**cTnT concentrtions**	Intraoperative concentrations positively correlate with outcome (51)	No correlation	Postoperative concentrations positively correlate with outcome (54)

*Abbreviations: cTnI – cardiac troponin I; cTnT – cardiac troponin T.

## NT-proBNP, BNP, and proBNP concentrations in animal studies and in patients with liver cirrhosis

BNP and NT-proBNP are among the most studied cardiac markers in patients with liver cirrhosis. Since the first study on this issue, performed by LaVilla et al (55) in 1992, numerous studies have examined the concentrations of natriuretic peptides in patients with liver cirrhosis. BNP concentrations were found to correlate with the severity of liver cirrhosis as assessed with the Child-Pugh and MELD scores and were higher in patients with liver cirrhosis than in the healthy population (56-61). In addition, BNP concentrations were higher in patients with liver cirrhosis than in those with NAFLD, and in patients with NAFLD than in healthy people (62). BNP concentrations also correlated with the degree of EV, presence of ascites, and collateral circulation, while they inversely correlated with plasma albumin concentration (55,60,62). Also, BNP concentrations were a predictor of six-month and one-year mortality in patients with liver cirrhosis (59,60,63). As for cardiac function, BNP concentrations correlated with interventricular septal and posterior heart wall thickness and worsening of diastolic function in terms of an increased diameter of the left atrium and a decreased ratio of early and late diastolic filling (E/A). They also correlated with a decreased ejection fraction in asymptomatic and symptomatic patients with liver cirrhosis (62,64). Furthermore, BNP concentrations were highly sensitive and specific in the early diagnosis of cirrhotic cardiomyopathy (65). In a retrospective study, BNP concentrations were found to be higher in liver cirrhosis patients with associated atrial arrhythmias than in patients without associated arrhythmias (66). Also, BNP concentrations above 300 pg/mL had a significant prognostic value for 90-day mortality and 90-day need for therapeutic paracentesis in patients with liver cirrhosis, and, due to their high specificity (>88%), could be included in prognostic algorithms in patients with liver cirrhosis (67).

Similarly to BNP, NT-proBNP is a predictor of mortality in patients with liver cirrhosis, as its concentrations are higher in patients with liver cirrhosis than in healthy population. NT-proBNP concentrations also correlate with the severity of liver cirrhosis as assessed with the Child-Pugh and MELD score in alcoholic and non-alcoholic liver cirrhosis and are higher in patients with decompensated than in those with compensated liver cirrhosis (40,45,68-73). NT-proBNP concentrations were shown to inversely correlate with the parameters of left ventricular diastolic function, such as the volume of the left atrium and E/A, in patients with liver cirrhosis and chronic liver disease, but due to low specificity, did not prove useful in screening of patients with cirrhotic cardiomyopathy (40,69,72,74). Also, NT-proBNP concentrations correlate with signs of hyperdynamic circulation (stroke volume, cardiac output, left atrial volume, reduction in systemic vascular resistance - decrease in systolic, diastolic, and mean arterial pressure) in patients with decompensated liver cirrhosis and do not correlate with a decreased ejection fraction as it is the case in heart failure. This is the reason for using NT-proBNP as a marker for titration of non-selecitve beta blocker therapy (71,75). NT-proBNP concentrations in patients with liver cirrhosis correlate with the length of QT interval and severity of pulmonary hypertension, and could serve as one of the indicators of pulmonary hypertension in patients with liver cirrhosis (70,76). NT-proBNP concentrations are several times higher in patients with ascites due to heart failure than in patients with ascites due to decompensated liver cirrhosis. However, in patients with liver cirrhosis, NT-proBNP concentrations were decreased after paracentesis (77,78). NT-proBNP concentrations above 101 pmol/mL in patients with liver cirrhosis are a good non-invasive predictor of EV (79).

Similarly to the other mentioned natriuretic peptides, proBNP correlates with the severity of liver cirrhosis in patients with heart diseases according to the Child-Pugh and MELD scores and also with intrahospital mortality in patients with liver cirrhosis (41). Furthermore, proBNP concentrations are higher in patients with cirrhotic cardiomyopathy than in patients with liver cirrhosis but without cirrhotic cardiomiopathy (80). Also, proBNP concentrations correlate with the degree of PH in patients with liver cirrhosis (81). A combination of BNP or NT-proBNP concentrations and echocardiographic measurements was a significant predictor of cardiac decompensation one year after TIPS (82).

An additional insight into the dynamics of natriuretic peptides in liver cirrhosis can be obtained from animal studies in which cirrhotic rats were compared with controls. In one of these studies, BNP was found to decrease the portal vein pressure, while natriuretic response to BNP administration was lower (83). In accordance, intravenous administration of low doses of BNP did not increase natriuresis in patients with liver cirrhosis and ascites (84). It is hypothesized that an endogenous antinatriuretic response causes a weaker natriuretic response to BNP stimulation in these patients (83,85). Also, mice overexpressing the BNP gene were more resistant to the development of fibrosis, probably because BNP inhibits liver fibrosis by inhibiting the activation of stellate cells in the liver (86). The relationship between BNP, NT-proBNP and mortality, Child-Pugh and MELD score, and left ventricular function is shown in Table 3.

**Table 3:** The correlation of cardiac brain natriuretic peptide (BNP) and N-terminal pro-B-type natriuretic peptide (NT-proBNP) concentrations with mortality, Child-Pugh score, model for end-stage liver disease (MELD) score, and left ventricular function

	Mortality	Child-Pugh score	Meld score	Left ventricular function
**BNP**	Correlation (55,59,60,66)	Correlation (56-60)	Correlation (61)	Inverse correlation (62,64)
**NT-proBNP**	Correlation (72)	Correlation (68,70-72)	Correlation (40)	Inverse correlation (40,69,72,74)

## NT-proBNP and BNP in liver transplantation

Elevated BNP concentrations correlate with diastolic dysfunction and overall mortality in patients who underwent a liver transplant (87). Lower NT-proBNP concentrations were observed after liver transplant compared with the pre-transplant period, and preoperative concentrations greater than 2000 pg/mL correlated with a higher incidence of CV events postoperatively. Although a decrease in NT-proBNP was recorded after transplantation, there was an increase in the mass of the left ventricle and a deterioration in the diastolic heart function (88). The authors of this retrospective study attributed NT-proBNP decrease mainly to a decreased volume overload, while the increase in the mass of the left ventricle and a deterioration in the diastolic heart function contradicted prior studies and need to be further evaluated (88). Preoperative BNP concentrations in patients who underwent liver transplant were an independent predictor of mortality in the intensive care unit and 180 days after surgery independent of the MELD score and showed an extremely high negative predictive value for mortality. In addition, BNP concentrations correlated with the length of mechanical ventilation after transplantation, the need for vasopressor application, and the need for renal function replacement (dialysis) (89). In a retrospective study by Moon et al, BNP concentrations, together with cTnI concentrations, were good predictors of 90-day mortality and graft loss in patients after liver transplant (50).

## H-FABP concentrations in patients with liver cirrhosis

Unlike previously mentioned markers of cardiac injury, H-FABP concentrations have not been extensively studied in patients with liver cirrhosis. H-FABP concentrations in patients with chronic liver disease (liver cirrhosis, alcoholic hepatitis, hepatitis B and C) were not found to significantly differ from those in healthy controls (90). The authors (90) assumed that since cTn concentrations were dependent on the degree of liver cirrhosis, H-FABP concentrations could be used as a marker of cardiac injury in patients with liver cirrhosis.

## Discussion

**Figure 1:**
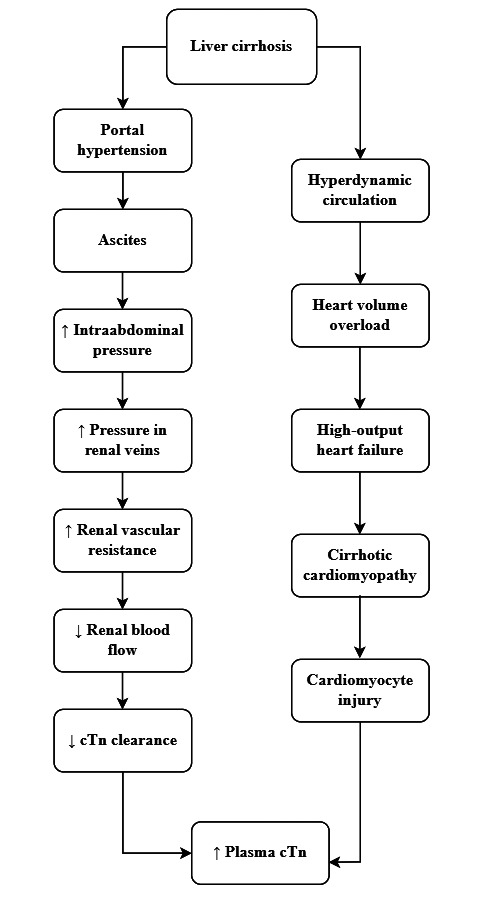
Proposed mechanism of cardiac troponin levels elevation in liver cirrhosis. cTn – cardiac troponin T.

According to most studies, cTnI and cTnT concentrations correlate with the clinical stage of liver cirrhosis. The mechanism of increase in troponin concentration in patients with liver cirrhosis has not yet been fully elucidated. As a result of increased blood flow resistance through the portal circulation and hypoproteinemia, patients with decompensated liver cirrhosis develop ascites, which increases the pressure on the organs in the abdominal cavity (91). A manifestation of ascites is an increased pressure on the renal veins, which can increase blood flow resistance and consequently decrease blood flow through the kidneys. Decreased blood flow through the kidneys, with increased water retention in the kidneys, because of a decreased intravascular volume then leads to cTn elevation (9,91). cTn are predominantly found in the blood in fragmented forms, and in physiological conditions, they are eliminated from the blood by renal filtration, which is why it is possible to measure cardiac markers in the urine (92). cTn concentrations are generally elevated in patients with chronic kidney disease (93,94). A decreased blood flow through the kidneys leads to increased cTn concentrations as a result of decreased clearance (Figure 1, left). A second proposed cause of the hyperdynamic circulation is stretching of the myocardium as part of the volume load (increase in end-diastolic pressure in the left ventricle), which can lead to an increased cTn release from the myocardium (84) (Figure 1, right).

In acute decompensation of liver cirrhosis, cTn are slowly eliminated from the blood and their concentration increases. Given that decompensation can be caused by numerous conditions, which can also increase the concentration of cTn in the blood (eg, infections), cTn concentrations might have a good prognostic value for in-hospital mortality, but not for long-term prognosis. We hypothesize that in the states of stabilization of decompensation, the majority of parameters that can affect the cTn concentration in the blood are normalized (or their influence is reduced). The concentrations measured in “stable” cirrhosis therefore could be a better prognostic factor for long-term prognosis.

Although the dynamics of the release of BNP and NT-proBNP into the circulation has been well investigated, the specificities of BNP and NT-proBNP elimination pathway have not yet been fully elucidated. The clearance of BNP depends on NPR-C concentration, and it takes place in different tissues. As in decompensated cirrhosis, there is an additional stimulus for BNP release, and due to endothelial dysfunction and reduced functional liver parenchyma, its elimination is reduced, which results in an increased BNP concentration in the blood (30). Given that different tissues affect the elimination of BNP from the blood, further research is needed to examine the potential influence of the remaining functional liver parenchyma on BNP concentration. Since the elimination pathways of BNP and NT-proBNP are different, we assume that their dynamics of elimination in patients with liver cirrhosis could be significantly different than in both the healthy population and patients with HF. As mentioned earlier, the concentrations of NT-proBNP and BNP are elevated in conditions of hyperdynamic circulation, which is probably one of the reasons for elevated values in patients with liver cirrhosis (75). An interesting theory would be that, in patients with liver cirrhosis, BNP is elevated partly as a compensatory reaction to liver injury since BNP hyperexpression in mice protects against liver fibrosis, and BNP in rats with liver cirrhosis had a significant effect on reducing the pressure in the portal vein despite a weaker natriuretic effect compared with healthy rats (83,86). NT-proBNP clearance might be independent of the liver function, and we believe it to solely depend on renal function. This characteristic should make it a superior marker of heart failure in patients with liver cirrhosis.

HFABP is a small globular molecule without polarity. We assume that HFABP clearance can be maintained in patients with liver cirrhosis, and its blood concentration does not have to differ from that in the healthy population (84,93-95). The only study to date examining the association between H-FABP and liver cirrhosis showed no correlation between liver cirrhosis and H-FABP concentrations. However, further research is needed to study the intricacies of H-FABP dynamics in patients with liver cirrhosis.

Although new criteria for cirrhotic cardiomyopathy are based on modern concepts of ventricular dysfunction, they are still not proven to have a prognostic value (96). We advocate that cardiac biomarkers such as cTn, BNP, and NT-proBNP are included in the diagnostic criteria of cirrhotic cardiomyopathy since these have prognostic value for long-term outcomes. Some other diagnostic criteria, such as stress-echocardiography, might have added prognostic value.

## Conclusion

Blood values of cTn in patients with acute decompensated liver cirrhosis correlate with the degree of decompensation and can have prognostic value only during hospitalization. We assume that their values in chronic decompensation of liver cirrhosis would be a better prognostic marker for a long-term follow-up because they are measured in a stable phase when there is a lower influence of other factors that can affect their concentration. Preoperative, intraoperative, and postoperative cTn values have been shown to be good predictors of mortality after liver transplantation. Since pretransplantation cTn concentrations have been shown to be a good predictor of liver graft rejection, they could be useful in selecting candidates for liver transplant or to optimize therapy in these patients.

NT-proBNP concentration is a good marker of hyperdynamic circulation, and in acute decompensated cirrhosis it correlates with the degree of decompensation. In chronic decompensation, NT-proBNP could be a better prognostic parameter that could indicate acute heart failure. Similar to cTn, preoperative values of NT-proBNP and BNP correlate with postoperative mortality after liver transplantation and have been shown to be predictors of CV incidents and cardiac dysfunction. Therefore, they could be used in the selection of candidates for liver transplantation.

HFABP has been shown to have normal values in patients with decompensated cirrhosis. We assume that its regular concentration is maintained by renal clearance despite the change in hemodynamics in acute decompensation. HFABP could therefore be an excellent marker for the diagnosis of ACS in patients with liver cirrhosis because normal concentrations in the blood have a high negative predictive value. Unlike HFABP, cTn is elevated in most of these patients.

In patients with liver cirrhosis and those in whom liver transplant is planned, after the decompensated form of the disease is stabilized, cardiac troponins (cTnI and cTnT) and NT-proBNP concentrations can identify the patients who have developed cirrhotic cardiomyopathy or who are at an increased risk of the disease. Elevated values of markers of cardiac injury point to the patients with a worse outcome and patients with liver transplants who are expected to experience graft dysfunction sooner. The patients with elevated values of these markers should be more frequently monitored by a gastroenterologist and undergo cardiology follow-up.

Further research is needed to evaluate the existing cardiospecific biomarkers and new ones, such as soluble suppression of tumorigenicity 2, galectin 3, cardiac myosin C binding protein, mid-regional pro atrial natriuretic peptide, and others. Further research should assess the effect of liver cirrhosis on the concentration of cardiospecific biomarkers.
